# Variability in Migration Routes Influences Early Marine Survival of Juvenile Salmon Smolts

**DOI:** 10.1371/journal.pone.0139269

**Published:** 2015-10-09

**Authors:** Nathan B. Furey, Stephen P. Vincent, Scott G. Hinch, David W. Welch

**Affiliations:** 1 Pacific Salmon Ecology and Conservation Laboratory, Department of Forest and Conservation Sciences, University of British ColumbiaVancouver, British Columbia, Canada; 2 Seymour Salmonid Society, North Vancouver, British Columbia, Canada; 3 Kintama Research Services Ltd., Nanaimo, British Columbia, Canada; Pacific Northwest National Laboratory, UNITED STATES

## Abstract

Variability in animal migratory behavior is expected to influence fitness, but few empirical examples demonstrating this relationship exist. The initial marine phase in the migration of juvenile salmon smolts has been identified as a potentially critical life history stage to overall population productivity, yet how fine-scale migration routes may influence survival are unknown. Large-scale acoustic telemetry studies have estimated survival rates of outmigrant Pacific salmon smolts through the Strait of Georgia (SOG) along the British Columbian coastline to the Pacific Ocean, but these data have not been used to identify and characterize fine-scale movements. Data collected on over 850 sockeye salmon (*Oncorhynchus nerka*) and steelhead (*Oncorhynchus mykiss*) smolts detected at an array in the Strait of Georgia in 2004–2008 and 2010–2013 were analyzed to characterize migration routes and link movements to subsequent survival at an array 250 km further along the marine migration pathway. Both species exhibited disproportionate use of the most eastern route in the Strait of Georgia (Malaspina Strait). While many smolts moved across the northern Strait of Georgia acoustic array with no indication of long-term milling or large-scale east-to-west movements, large proportions (20–40% of sockeye and 30–50% of steelhead) exhibited a different behavior, apparently moving in a westward or counterclockwise pattern. Variability in migratory behavior for both species was linked to subsequent survival through the Strait of Georgia. Survival for both species was influenced by initial east-to-west location, and sockeye were further influenced by migration timing and duration of time spent near the northern Strait of Georgia array. Westward movements result in a net transport of smolts from Malaspina Strait to the Strait of Georgia, particularly for steelhead. Counterclockwise movements may be due to the currents in this area during the time of outmigration, and the higher proportion of steelhead smolts exhibiting this counterclockwise behavior may reflect a greater exposure to wind-altered currents for the more surface-oriented steelhead. Our results provide an empirical example of how movements can affect migration survival, for which examples remain rare in movement ecology, confirming that variability in movements themselves are an important part of the migratory process.

## Introduction

An organism’s movements directly impacts its ecology, as changes in spatial locations over time influence interactions among conspecifics, competitors, predators, and/or resources [1]. Migrations are a unique subset of directed movements that are observed across taxa including mammals, birds, reptiles, invertebrates, and fish [2,3]. The movement trajectory or route defines the landscapes traversed and consequently the conditions experienced [1]. In theory, migration routes should be optimized to improve probability of survival, but routes can still vary considerably among individuals [4–6], resulting in differing energetic costs and conditions being experienced [7,8], presumably due to either suboptimal behavior or individual-specific optima [9]. At present, few empirical examples relate variability in movements to future fitness or survival to confirm the importance of movements to an organism’s ecology [1,10]. Characterizing the variability in the specific routes utilized and relating this individual variability to survival is needed to better understand the migratory process.

Among migratory fish, anadromous salmon are among the most studied due to their value. Juvenile salmon smolts enter marine waters after rearing in freshwater, and survival during the early coastal migration has been suggested to be size or growth dependent [11,12] and influence overall population productivity [11–14]. Migratory behavior in the early marine environment, however, remains understudied [15,16], and there has been a call to assess the impacts of migration patterns on smolt survival [17]. The current paradigm for Pacific smolt movements in estuarine and coastal waters describes linear migratory paths [18,19], but this paradigm has largely been constructed on population-level sampling using purse seine of unmarked individuals during the migratory period and inferring the migration biology from the size and relative abundance of individuals in the catches. The proposed movement patterns have not been verified directly. Therefore, there is a need to characterize migration routes of individuals and to identify factors that result in variability in migratory experience.

For many Pacific salmonids, including those of the productive Fraser basin, the Strait of Georgia, a narrow semi-enclosed marine sea, represents an important component of the migratory corridor. Of these species, smolts across populations of both sockeye salmon (*Oncorhynchus nerka*) and steelhead trout (*Oncorhynchus mykiss*) are thought to move quickly through the Strait of Georgia and then along the continental shelf before eventually reaching offshore feeding grounds, unlike other species that can remain in the Strait of Georgia for extended periods [17,20–24]. Coastal migrations are assumed to be a result of using local cues to assist in migratory navigation (reviewed in [25]). Large-scale acoustic networks have been used to describe migration rates and survival in coastal waters for outmigrant juvenile salmon [17,22,26–29], but the fine-scale migration routes used in marine waters have largely remained undescribed nor has their relationship with survival been assessed.

The characterization of movement has become increasingly possible with the advent and continued development of biotelemetry [30]. For small-bodied fishes such as smolts, acoustic telemetry in particular provides a balance between tag size and detection efficiency. Fine-scale arrays have been used to track movements of individual fishes using triangulation methods [31–33], but generating these arrays at spatial scales large enough to determine marine migration routes of smolts would be cost-prohibitive. Conversely, large-scale telemetry networks, such as the Pacific Ocean Shelf Tracking (POST; [34]) system and the Ocean Tracking Network [35,36], utilize multiple arrays spaced at greater distances, but are generally used for broad-scale estimates of migration rates and survival rather than characterizing specific migration routes (i.e., [22,29]). These networks, however, are often made up of “curtains” or arrays of many individual receivers to help ensure detection at specific checkpoints along movement corridors [37], and therefore it is possible that more detailed information on behavior could be obtained by examining data within a single array rather than simply among arrays.

**Fig 1:**
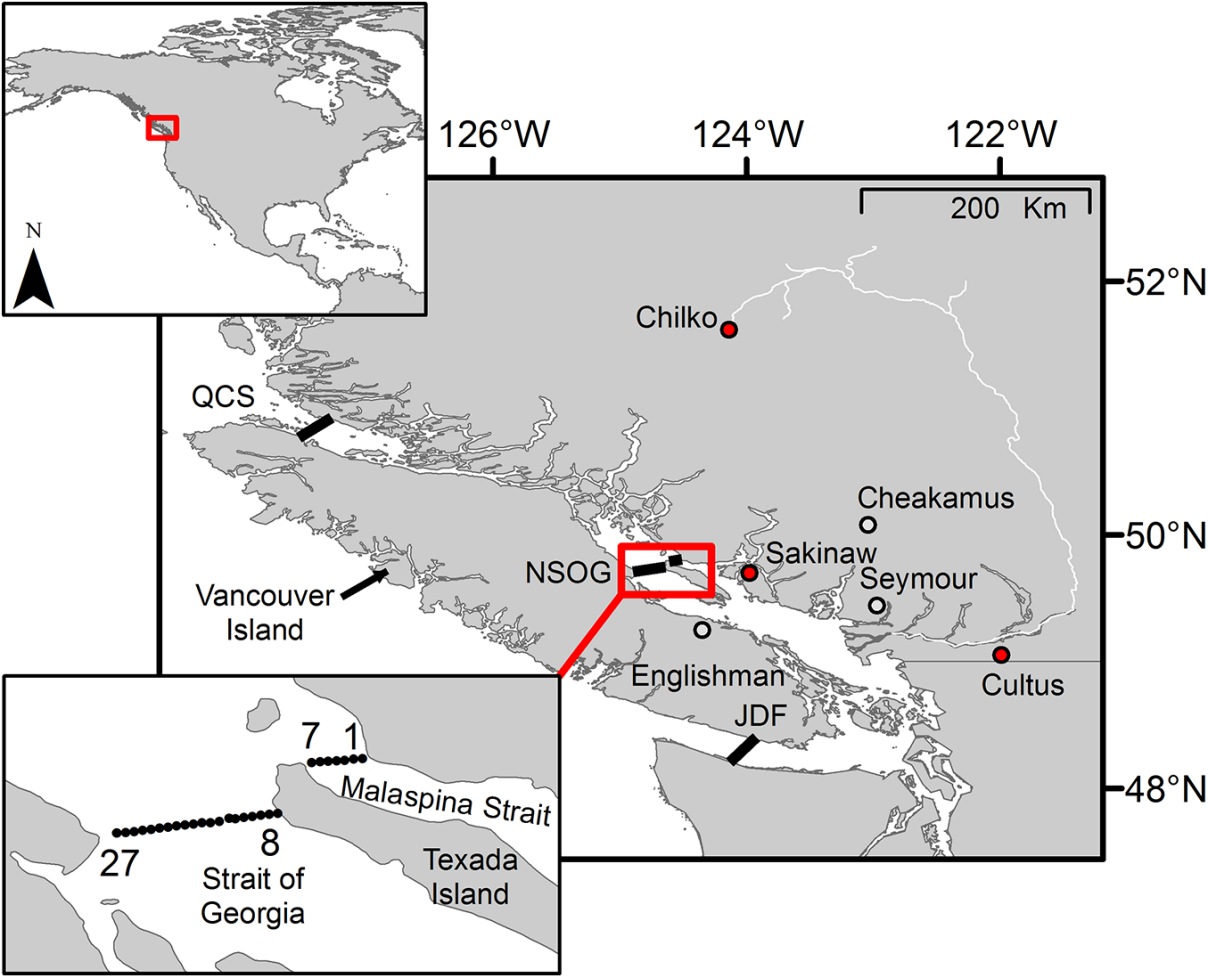
Study area, and acoustic telemetry infrastructure, and natal populations. The location of acoustic telemetry receiver arrays (black lines) and natal freshwater rearing areas for sockeye salmon (red dots) and steelhead (grey dots). The bottom-left inset depicts the arrangement of acoustic telemetry receivers within the northern Strait of Georgia (NSOG) array. First (1) and last (27) receivers are labeled to reference the numbering scheme used in analyses.

The current study aims to determine the migratory routes used by juvenile sockeye and steelhead smolts in the Strait of Georgia, and to determine the influence of individual variability in movements on survival. Using acoustic telemetry data collected across populations from 2004–2013 [17,22,26–29] (Table 1), we examined detections at a single large-scale array: the northern Strait of Georgia (NSOG; Fig 1). We assessed initial east-to-west distributions of >850 smolts arriving at this array, the direction and magnitude of subsequent movements, and survival to the next array at Queen Charlotte Strait (QCS), located ~250 km farther along the migration path (Fig 1).

**Table 1:** Summary of sockeye salmon and steelhead smolts tagged with acoustic telemetry transmitters and tracked in the Strait of Georgia. The number of smolts detected at the Northern Strait of Georgia (NSOG) array and subsequently at the Queen Charlotte Strait (QCS) array are given, as well as the number of each migratory route (linear, counter, and clockwise) as defined in the Materials and Methods. Populations are labeled as hatchery origin (H), wild (W), or a mix of both (H/W).

Year	Species	Population	Mean FL ± SD (mm)	Number released	Number detected at NSOG	Number detected at QCS	Number linear	Number counter	Number clockwise	Data sources
2004	sockeye	Cultus (H)	178 ± 13	100	34	13	13	1	0	[17,22,29]
2004	sockeye	Sakinaw (H)	193 ± 15	97	38	16	16	4	1	[17,22]
2005	sockeye	Cultus (H)	177 ± 14	378	44	22	17	8	4	[17,22,29]
2005	sockeye	Sakinaw (W)	191 ± 12	47	13	3	2	3	3	[17,22]
2006	sockeye	Cultus (H)	178 ± 9	200	54	30	26	6	3	[17,22,29]
2006	sockeye	Sakinaw (H/W)	206 ± 14	136	40	15	12	7	6	[17,22]
2007	sockeye	Cultus (H)	182 ± 11	319	107	64	58	12	4	[17,22,29]
2010	sockeye	Chilko (W)	130 ± 4	199	25	4	3	4	1	[26]
2011	sockeye	Chilko (W)	133 ± 7	443	54	24	23	2	0	[26]
2012	sockeye	Chilko (W)	123 ± 4	386	60	18	19	7	3	[26]
2013	sockeye	Chilko (W)	123 ± 2	432	184	48	42	32	13	[26]
Totals sockeye				2737	653	257	231	86	38	
2004	steelhead	Cheakamus (W)	184 ± 17	51	26	10	7	7	2	[17,22,27,28,38]
2004	steelhead	Englishman (W)	174 ± 17	67	41	10	8	9	3	[17,22]
2005	steelhead	Cheakamus (W)	178 ± 14	49	26	13	10	5	1	[17,22,27,28,38]
2005	steelhead	Englishman (W)	159 ± 15	43	20	5	4	2	2	[17,22]
2006	steelhead	Englishman (W)	169 ± 12	50	34	12	9	10	2	[17,22]
2006	steelhead	Seymour (H)	207 ± 16	50	5	1	0	3	0	[39]
2007	steelhead	Cheakamus (H)	183 ± 11	100	12	3	3	1	0	[17,22,28,38]
2007	steelhead	Seymour (H)	186 ± 13	60	6	1	1	1	0	[17,39]
2008	steelhead	Cheakamus (H/W)	180 ± 12	198	55	20	17	14	1	[38]
2008	steelhead	Seymour (H)	184 ± 14	60	9	4	4	2	0	[39]
Totals steelhead				728	234	79	63	54	11	

## Materials and Methods

### Acoustic telemetry studies

We examined marine movement patterns from acoustic telemetry data for three sockeye populations (Cultus Lake [2004–2007], Sakinaw Lake [2004–2007], and Chilko Lake [2010–2013]), and three steelhead populations (Englishman [2004–2006], Seymour [2006–2008], and Cheakamus [2004–2005 and 2007–2008]) whose natal streams (or hatcheries) lie in watersheds of the lower Strait of Georgia (Fig 1 and Table 1). These populations share a similar early marine migration during the late spring and/or early summer (release dates of tagged smolts occur mid-April to early-June), in which smolts migrate to the north through the Salish Sea region (Fig 1) to the open Pacific [22]. For sockeye, body size and rearing origin differ between Chilko and Cultus sockeye. Chilko sockeye smolts are from a wild and large population (up to 40 million smolts outmigrate each year) for which tagged fish are generally 120–130 mm FL. In comparison, Cultus sockeye is an endangered population and the smolts we tagged were reared under hatchery conditions as part of a conservation program to rebuild this population, and all tagged individuals were larger in comparison (generally 175–190 mm FL). In addition, Englishman steelhead are the only population in the current study that originates on Vancouver Island rather than the British Columbia mainland.

For all studies, smolts were captured in natal freshwater systems or collected from a hatchery, and then implanted with either VEMCO (Halifax, NS) 69 kHz V7 (Chilko sockeye) or V9 (all other populations for both species) acoustic tags through a small abdominal incision along the ventral side of the body. Further details regarding surgeries can be found within references cited in Table 1.

### Acoustic receiver arrays

This study focuses on the detections of tagged smolts at the northern Strait of Georgia (NSOG) array (Fig 1), which was installed by the Pacific Ocean Shelf Tracking project [34] and subsequently maintained by the Ocean Tracking Network [35]. The NSOG array spans the Strait of Georgia and Malaspina Strait, and contains 26 separate acoustic telemetry receivers which are spaced at approximately 800 m intervals (Fig 1), with the 7^th^ and 8^th^ receivers separated by ~8km on either side of Texada Island (Fig 1). A 27^th^ receiver was also part of the array for some of the studies, but only a total of 4 smolts were ever detected on this receiver. Due to the geometry of this array, it is possible to characterize the east-to-west distribution in detections of smolts as they approach from the south, and then examine their subsequent behaviors. Smolts could then be subsequently detected at the Queen Charlotte Strait (QCS) array approximately 250 km to the northwest as they exit the Strait of Georgia (Fig 1). Detections at the QCS array can be utilized to determine subsequent survival of smolts initially detected at the NSOG array. Depending on the year, arrays were made up of VEMCO 69 Khz VR2, VR3, or VR4 receivers. Smolts could also be detected on the western Juan de Fuca Strait array (JDF; Fig 1), but < 2% of smolts were detected here, and thus detections on this array were not analyzed.

### Detection data

Large-scale acoustic monitoring systems such as the NSOG array generate large quantities of detection data, much of which is redundant (in the sense that multiple detections of the same animal at closely spaced time intervals does not constitute new information but merely verification of occurrence at a particular time). Analyses were based on ‘detection sequences’ rather than just ‘detections’, allowing detections be grouped in order to represent distinct occurrences at the array. We define a detection sequence as one or more detections of a unique tag (i.e., individual smolt) on a receiver array such that 1) consecutive detections are less than one hour apart, and 2) consecutive detections occur within two receivers’ distance of the previous detection (≤1.6 km). Individual detection sequences can be summarized by the following characteristics: 1) the date, time, and receiver position number (or distance in km) of the first detection, and 2) the date, time, and receiver position of the last detection.

### Data analysis

The initial distribution of smolts from each species was examined by determining the number of smolts whose first detection sequence was initiated at each of the 27 receivers in the NSOG array. To test for disproportional use of Malaspina Strait (receivers 1–7, spanning the coastal ocean between the British Columbia mainland and Texada Island) versus the Strait of Georgia (receivers 8–27, spanning the coastal ocean between Texada Island and Vancouver Island; Fig 1), a proportional test was conducted for each species, weighted on the relative width (number of receivers in the array) of each strait. To examine how distribution might be influenced by release point (natal location), the same analysis was conducted using only Englishman River steelhead (the only population to originate on Vancouver Island).

The difference in receiver position between the first and last detection within the initial detection sequence at NSOG gives insight as to whether the smolt was moving directly through (i.e., perpendicular to) the array, or moving at an angle towards the east or west. The difference in receiver position between the last and first detections within the initial detection sequence was calculated for each smolt, such that movements to the west resulted in a negative value, and movements to the east resulted in a positive value. For each species, a Mann-Whitney U test determined if the difference in receiver position significantly differed from zero.

By comparing the locations of the first detection sequence and second detection sequence, a smolt’s migratory experience or “migratory route” was defined. A “counterclockwise” migration route occurred if the second detection sequence occurred to the west of where the first ended, and a “clockwise” migration route occurred if the second detection sequence occurred to the east of where the first ended. If the second detection sequence occurs not on the NSOG array but at the QCS array, we define the smolt as not undergoing detectable east-west movements in the immediate area of the NSOG array. Although a smolts’ movements elsewhere between the NSOG and QCS arrays are unknown, for the purpose of characterizing the variability in movements observed, we define this detection history as a “linear” migration route. Linear routes also included instances when the second detection sequence began at the same receiver as the last detection of the first sequence. The proportion of each migratory route (linear, counterclockwise, and clockwise) was calculated for each release group (year and population combination) for each species, as it was expected that the routes experienced within a release could be correlated. Analysis of variance (ANOVA) was used for each species to compare proportions among migratory routes. In addition, ANOVAs were weighted by the number of individuals within each release group that exhibited one of the three routes (i.e., as release groups varied greatly in sample size (Table 1). When the ANOVA was significant, pair-wise comparisons among all migration routes were made with Tukey’s honestly significant differences (HSD).

Once migratory routes were defined, the scale of movements along the NSOG array were calculated. East-to-west differences (Δx) between the end of the first and the beginning of the second detection sequences (Δx_12_) and similarly between the second and third (Δx_23_) were calculated as the difference in the number of receivers between these sequences (two adjacent receivers represents a Δx of 0.8 km; if the subsequent detection is to the east of the original Δx = 0.8 and if the subsequent detection is to the west of the original Δx = -0.8 km). If the second detection sequence began at the location of the first, a Δx of zero was assigned. In addition, for movements spanning Texada Island along the NSOG array (Fig 1), Δx was adjusted by 8.0 km (positive for eastward directions, negative for westward directions) as the mean distance between each receiver in the array (804 m) is approximately one-tenth that of the receivers on either side of the island (7.9 km). One-sample Wilcoxon signed-rank tests were used to determine if the mean Δx_12_ or Δx_23_ for each species differed significantly from zero to determine if an overall bias in directional movement was present. Mann-Whitney U tests examined if Δx_12_ or Δx_23_ was significantly different between the two species, or if Δx_12_ differed among sockeye populations. Kendall’s τ correlations were assessed between Δx_12_ and the duration of time between the first and second detection sequences. Assumptions of parametric tests were assessed visually, and if assumptions were not met non-parametric equivalents were used. For all hypothesis-based testing, we specified α = 0.05 to determine significance.

For each smolt that was detected at the NSOG array, its subsequent apparent survival (hereafter referred to as ‘survival’) was assessed using detections at the Queen Charlotte Strait (QCS; Fig 1) array lying ~250 km to the northwest of the NSOG array and well beyond the exit from the Strait of Georgia. Thus smolts that had any detections at the QCS array were considered survivors, and all others are assumed mortalities. Estimates of detection efficiency for the marine arrays for the tag types used (V7 and V9) range from 60–90%, respectively [29]. For each species, two sets of binomial general linear models (GLM) were constructed to relate movement and migration experience to subsequent survival to QCS (S_QCS_), with the first focusing on metrics pertaining to the entry of smolts to the NSOG array using all smolts that were detected at the array, and the second focusing on lateral east-west movements after initial NSOG detection using all smolts with at least two NSOG detection sequences. Constructing two sets of GLMs allowed for maximizing sample size, as the number of smolts with two or more distinct detection sequences (and therefore able to examine east-west movements for) is substantially smaller than the total number of smolts detected at the NSOG array (Table 1). In the first set of GLMs, explanatory variables included the receiver position of the initial NSOG detection (P_Initial_), the date of the initial NSOG detection, FL, natal population, and year of release. The interactions between natal populations and both Julian date and initial receiver position were also included. For the second set of GLMs, explanatory variables included the difference in receiver position between the first two NSOG detections (Δx_12_), the time between the first two NSOG detections (“duration”), FL, natal population, and year of release. The interactions between natal populations and both the difference in receiver position and the duration between detection sequences were also included. Year of release and FL were not included as variables for both sets of sockeye models as these variables were highly correlated with natal population. For the second set of steelhead models, only 6 Seymour smolts had at least two detection sequences at the NSOG array, and were removed from analyses.

All continuous variables in both sets of GLMs were centered by subtracting the mean from each value and dividing by two-times the standard deviation [40]. For each set of models, an all-subsets regression was conducted using the ‘MuMIn’ package [41] in R [42], and all models were ranked based on the corrected Akaike Information Criterion (AICc). For each model, the change in AICc from the top-ranked model (ΔAICc) was also calculated. Model coefficients, and 95% confidence intervals of these coefficients, were calculated using a model averaging approach across all models for which ΔAICc ≤ 2 [43]. Model fit was further assessed using AICc weight (W_i_), R^2^, adjusted R^2^, and log-likelihood. For variables whose 95% confidence intervals of the model-averaged coefficient did not contain zero, prediction plots were generated to visualize the modeled effect of the variable(s) on S_QCS._ Because relating migration metrics to survival can be confounded by migration rate (i.e., smolts with the same daily survival rate will exhibit different net survival to QCS if they take different amounts of time to reach QCS), we assessed the correlation (Pearson correlation coefficient) between these variables and the time taken to reach the NSOG from the previous array (entrance to the SOG). In addition, we assessed the correlation between each of these variables and the time taken to reach QCS among survivors.

## Results

Across the releases used in this study, 3017 sockeye and 728 steelhead smolts were tagged and released (Table 1). Of these releases, 582 sockeye and 219 steelhead smolts were detected at least once on the NSOG array, and 146 sockeye and 68 steelhead had multiple detection sequences at NSOG. In addition, 78 sockeye and 49 steelhead had three or more detection sequences at NSOG (Table 1). The mean date of arrival at the NSOG array across all releases was May 27 for both sockeye (SD = 16 days) and steelhead (SD = 11 days). Only 28 sockeye (0.9%) and 14 steelhead (1.9%) were detected using the westward route through the Strait of Juan de Fuca rather than northward through the Strait of Georgia (i.e., through the NSOG array; Fig 1).

### Initial Arrival

**Fig 2:**
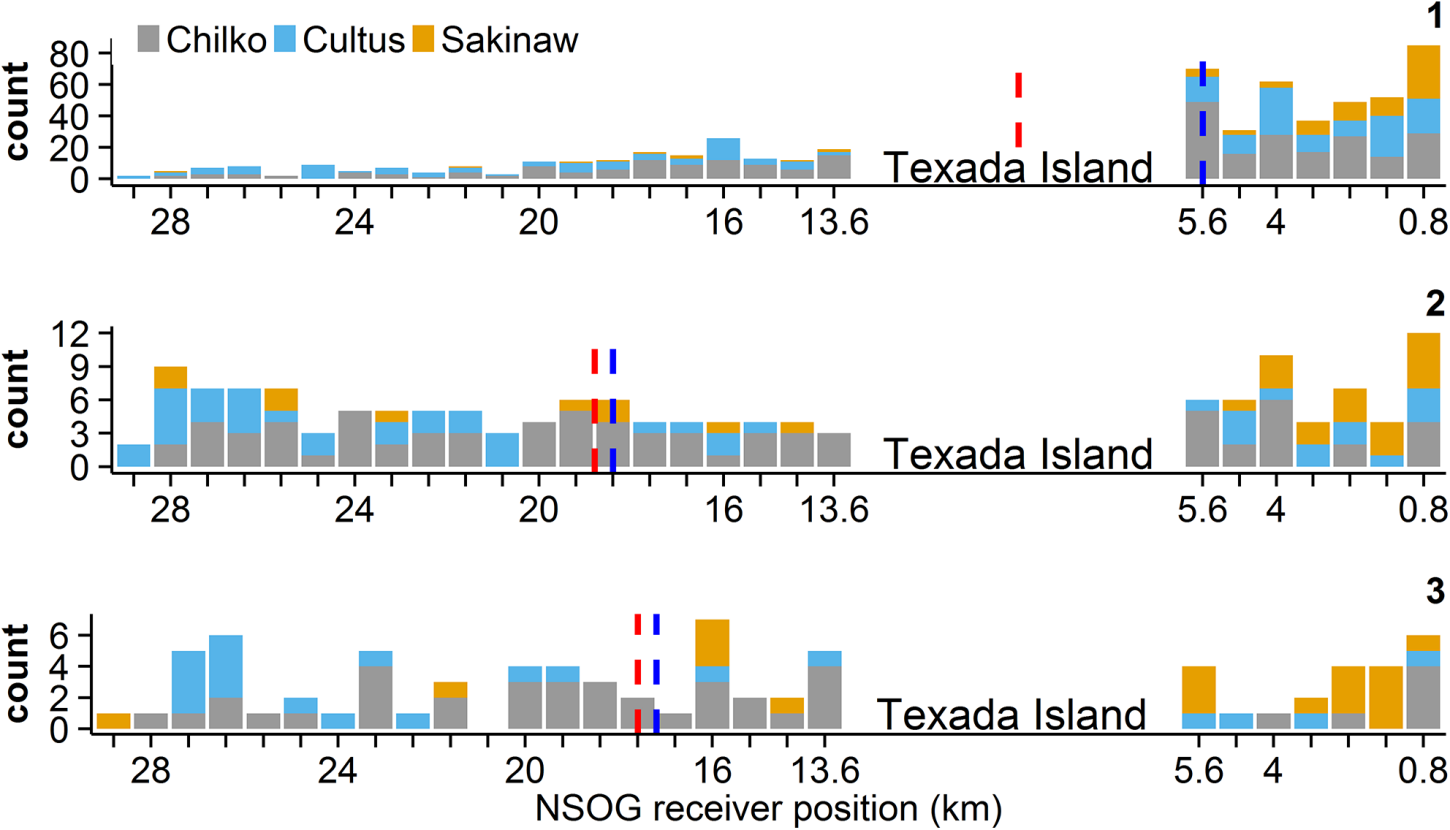
The distribution of sockeye salmon smolts on the northern Strait of Georgia (NSOG) array for the first (1), second (2) and third (3) detection sequences. Stacked colors indicate populations. Receiver positions represent those shown in the inset of Fig 1, and the gap between receivers 7 (5.6 km) and 8 (13.6 km) indicate the position and relative width of Texada Island. Dashed vertical lines indicate the mean (red) and median (blue) positions of sockeye smolts for each detection sequence. Note that the mean position of the first detection sequence was directly between Malaspina Strait (receiver positions 1–7) and Strait of Georgia (receiver positions 8–27).

**Fig 3:**
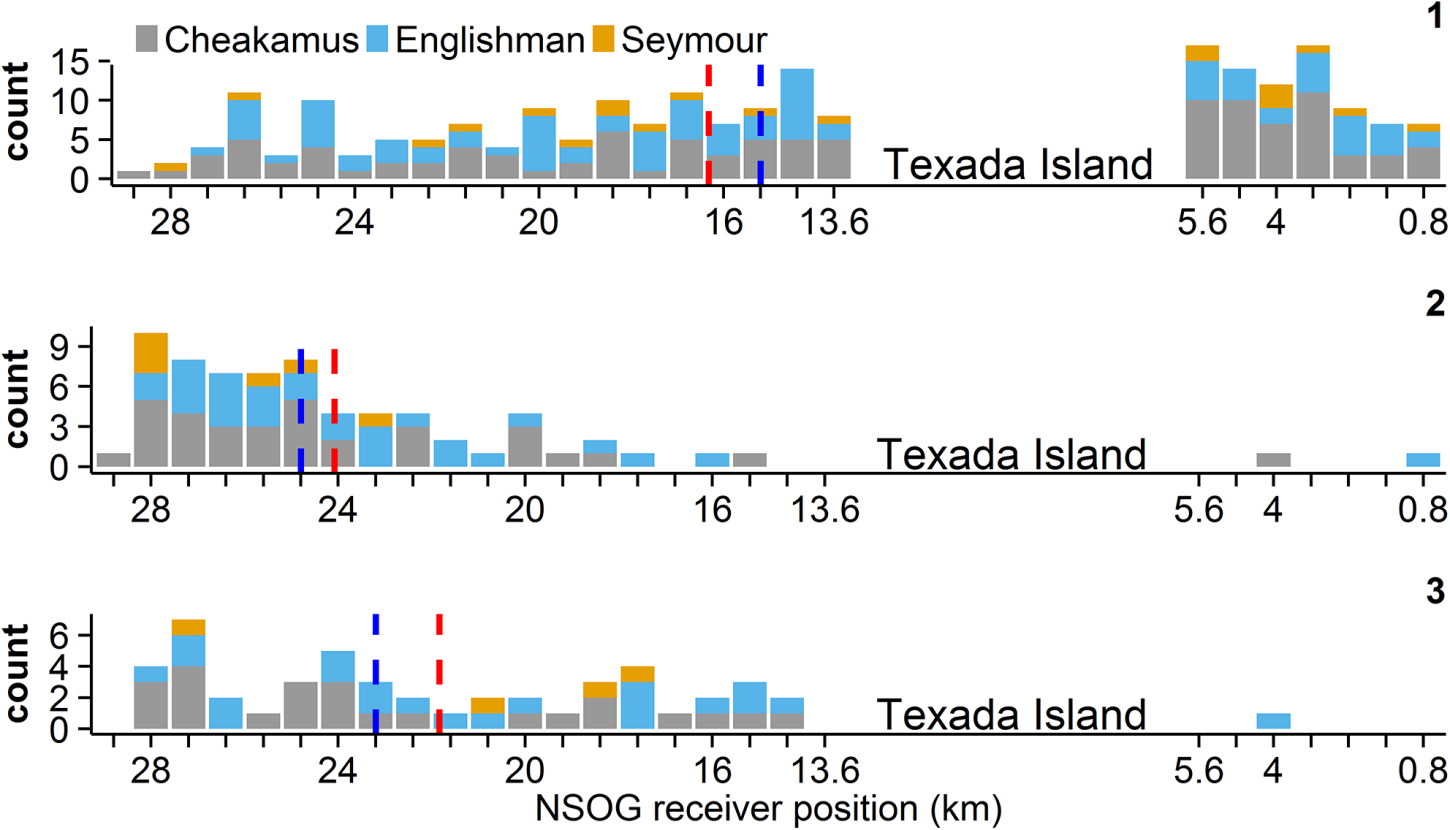
The distribution of steelhead smolts on the northern Strait of Georgia (NSOG) array for the first (1), second (2) and third (3) detection sequences. Details as in Fig 2.

The majority of sockeye (~66%) arrived to the NSOG array via Malaspina Strait between Vancouver Island and the British Columbia mainland (Fig 2). Significantly more sockeye arrived via Malaspina Strait than the Strait of Georgia when accounting for each strait’s width (p < 0.0001). The median initial position at the array for steelhead was in the Strait of Georgia (Fig 3), and although only 38% of steelhead arrived via Malaspina Strait, this use was disproportionately higher than expected given the strait’s width (p < 0.001). The proportion of Englishman steelhead, the only population between the two species found on Vancouver Island (Fig 1), arriving via Malaspina Strait was only 30%, which was not more than the use of the Strait of Georgia (p = 0.34).

The difference in receiver position between the first and last detections within the initial detection sequence was significantly different from zero for both sockeye (p < 0.001) and steelhead (p < 0.001). This difference in receiver position within the first detection sequence was not significantly different between sockeye (0.3 km to the west ± 0.3 km [mean ± SD]) and steelhead (0.4 km to the west ± 0.2 km) smolts (p = 0.55).

### Lateral Movements

**Fig 4:**
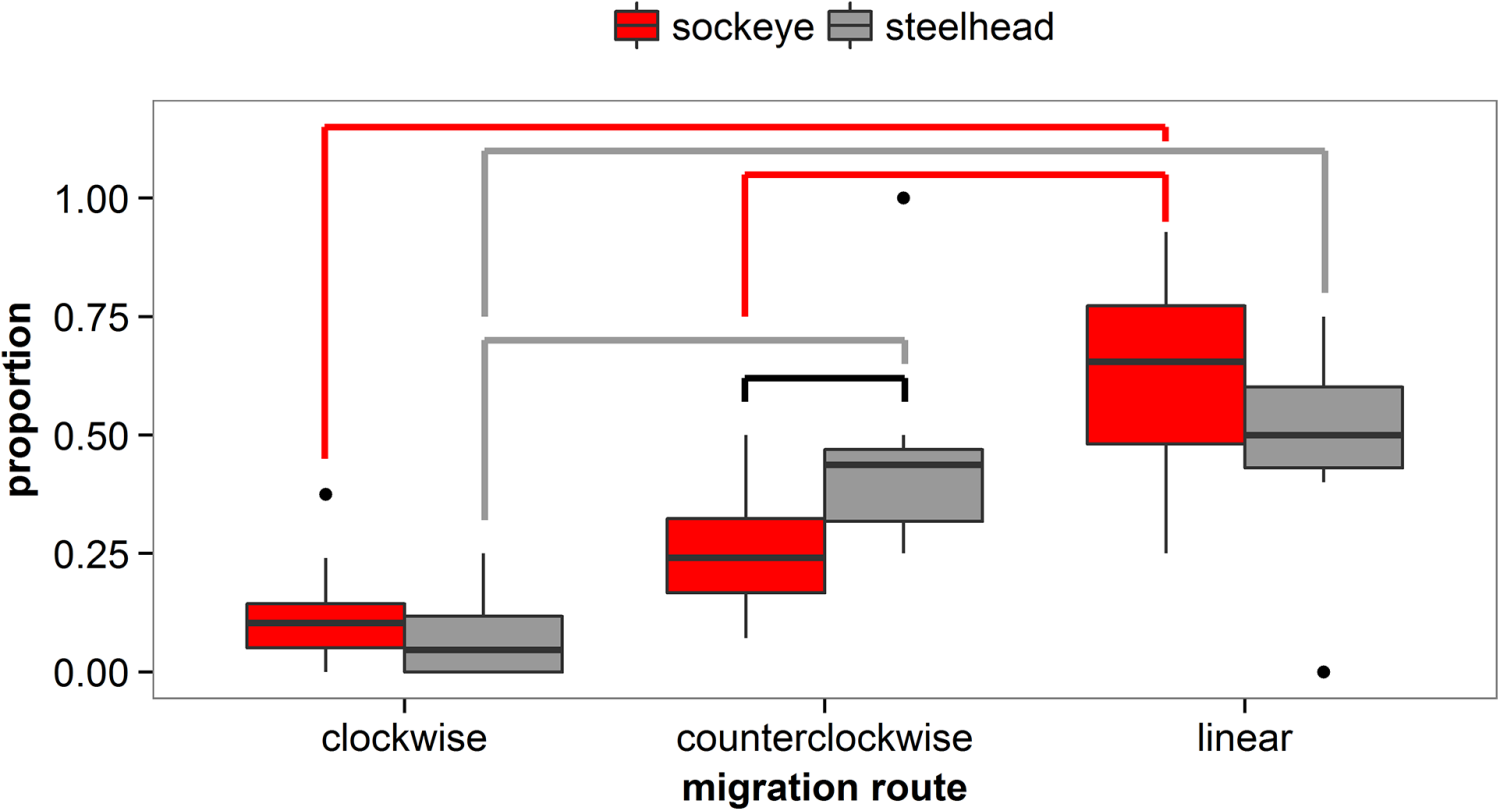
Boxplots of the proportion of sockeye salmon and steelhead smolts exhibiting linear, counterclockwise, and clockwise migration behaviors. Box limits represent the 25^th^ and 75^th^ percentile values. Lines indicate significant differences in proportions between migration routes for sockeye (red; Tukey HSD), and steelhead (grey; Tukey HSD), or between species within a migration route (black; t-test).

The proportional use of migratory routes (“clockwise,” “counterclockwise,” and “linear”) among release groups of sockeye smolts differed significantly (p < 0.0001; Fig 4). Pairwise comparisons determined that the proportion of sockeye smolts taking linear routes (as measured by the NSOG array) was greater than the proportion of smolts moving both counterclockwise (p < 0.0001) and clockwise (p < 0.0001). There was no significant difference, however, between the proportion of sockeye smolts taking counterclockwise and clockwise routes (p = 0.14).

For steelhead, there were also significant differences among migration routes experienced (p < 0.0001; Fig 4), with smolts taking counterclockwise and linear routes more often than clockwise (p < 0.0001 and p < 0.0001, respectively). There was no difference between the proportions of counterclockwise and linear smolts (p = 0.66).

A significantly smaller mean proportion of sockeye smolts (0.25 ± 0.14 [mean ± SD]) exhibited a counterclockwise migration route than steelhead (0.41 ± 0.10 [mean ± SD]) smolts (p = 0.02; Fig 4). No differences were found between sockeye and steelhead in the mean proportion of linear (p = 0.12) and clockwise (p = 0.60) migrants.

Overall, the displacement in receiver position between the first and second detection sequences (Δx_12_) of sockeye smolts along the NSOG array was westward (4.8 km to the west ± 8.8 km [mean ± SD]) and significantly different from zero (p < 0.0001), with the median receiver position of the second detection sequence located in the Strait of Georgia (Fig 2) and the distribution of smolts between the two straits non-uniform (p = 0.04). No significant differences in the westward displacement of sockeye smolts were found among populations (p > 0.05). Of the 146 sockeye smolts having multiple detection sequences at NSOG, 37 (25%) either spanned Texada Island or had a displacement of at least 8 km (10 receivers) between their first and second detection sequences. The mean duration between first and second detection sequences of sockeye was 69 hours (SD = 98 hours). A significant positive relationship was found between the duration between first and second detection sequences and the change in receiver position (p < 0.0001; S1 Fig).

Similarly, Δx_12_ of steelhead smolts along the NSOG array also indicated a westward displacement (9.6 km to the west ± 9.6 km [mean ± SD]) that significantly differed from zero (p < 0.0001), and the mean duration between first and second detection sequences was 72 hours (SD = 71 hours). The overall magnitude of movements of steelhead was greater than those of sockeye (p < 0.001), and the second detection sequence for the majority of steelhead occurred in the Strait of Georgia (Fig 3). Of the 68 steelhead to have multiple detection sequences at NSOG, 36 (53%) had westward displacements that either spanned Texada Island or at least 8 km distance. A significant positive relationship was found between the duration between the first two detection sequences and the absolute difference in receiver positions (p < 0.0001; S1 Fig).

**Fig 5:**
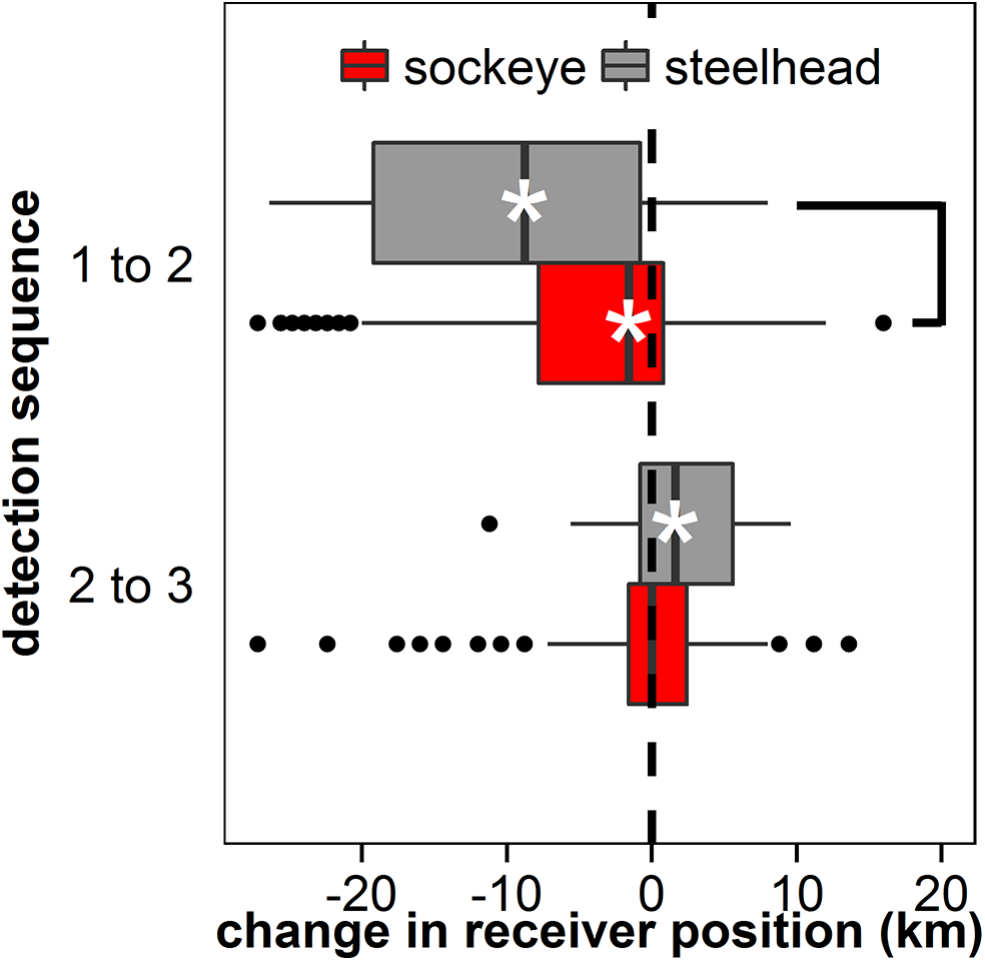
Differences in position (km) of sockeye (red) and steelhead (grey) smolts at the northern Strait of Georgia (NSOG) array between detection sequences. (1) Between first and second detection sequences, and (2) between second and third detection sequences. Negative values indicate westward movements, and positive values indicate eastward movements. Asterisks indicate significant differences from zero (Wilcox-signed rank test). Lines indicate significant differences in change in position between species (black; Wilcox signed-rank test). As described in the Materials and Methods, the change in receiver position for any movements that spanned Texada Island were adjusted by 8 km.

**Fig 6:**
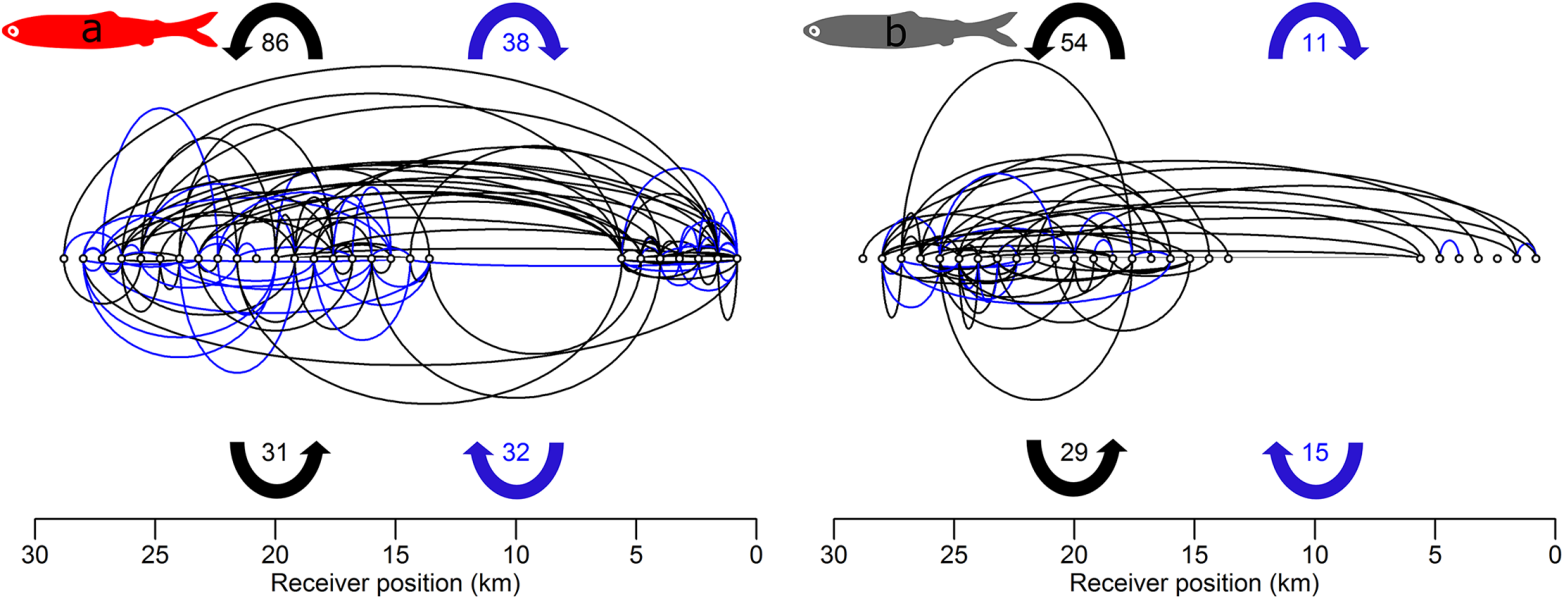
Visual representation of lateral movements taken by sockeye (a) and steelhead (b) smolts. Open circles represent the 27 receivers at the northern Strait of Georgia (NSOG) array. Lines indicate counterclockwise (black) or clockwise (blue) movements by individual smolts between either the first and second detection sequence (above receivers) or between the second and third sequence (below receivers). Line length is set equal to a distance travelled assuming each smolt swims continuously at 1 BL/s for the entire time duration between detection sequences. This distance (half of an ellipse’s perimeter) was used to estimate the vertically-oriented radius of the ellipse for plotting purposes using Ramanujan’s first approximation [44]. Vertical scale (not shown) is equal to horizontal scale. Ellipses were drawn using the plotrix package [45] in R [42]. Smolts exhibiting linear migration routes (see Materials and Methods) are not shown.

The difference in receiver position between the second and third detection sequences (Δx_23_) for sockeye (0.6 km to the west ± 6.8 km [mean ± SD]) was not significantly different from zero (Wilcoxon signed-rank test; V = 976, P = 0.83). For steelhead, this difference from zero was significant (1.5 km to the east ± 4.4 km [mean ± SD]; p = 0.02; Fig 5), but was not significantly different compared to sockeye (p = 0.07). By the third detection sequence, the majority of smolts for both species (72% for sockeye and 98% for steelhead) were found in the Strait of Georgia rather than Malaspina Strait (Fig 2, Fig 3), with distribution of sockeye not different from uniform across the array (p = 0.65), while steelhead were found almost exclusively in the Georgia Strait, and significantly so (p < 0.0001). The lateral movements of individual smolts (i.e., initial positions and Δx_12_ and Δx_23_) of both species are visualized in Fig 6.

The duration between first detection at NSOG array and first detection at QCS array was similar for both sockeye (12.5 ± 5.2 days [mean ± SD]) and steelhead smolts (12.4 ± 4.1 days [mean ± SD]). This duration for sockeye was not influenced by migration route (p = 0.10). Steelhead smolt residence was significantly influenced by migration route (p < 0.001), as duration was 4.3 days shorter for linear migrants (11.2 ± 3.4 [mean ± SD]) than counterclockwise (15.5 ± 4.1 days [mean ± SD]) migrants (p < 0.001).

### Survival

**Fig 7:**
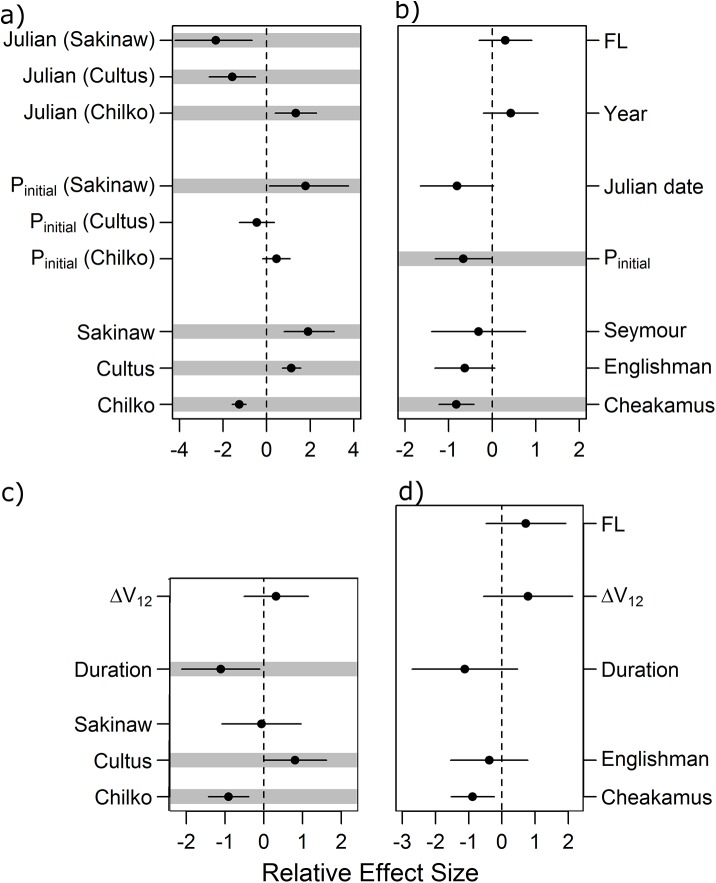
Relative effect sizes of model-averaged coefficients from general linear models (GLMs). Grey bars indicate variables for which the 95% confidence interval of the effect size does not include zero. As described in the Materials and Methods, separate models for each species were created using all smolts detected at the NSOG array (sockeye “a”, steelhead “b”), and for smolts detected for multiple periods at the NSOG array (sockeye “c”, steelhead “d”).

**Fig 8:**
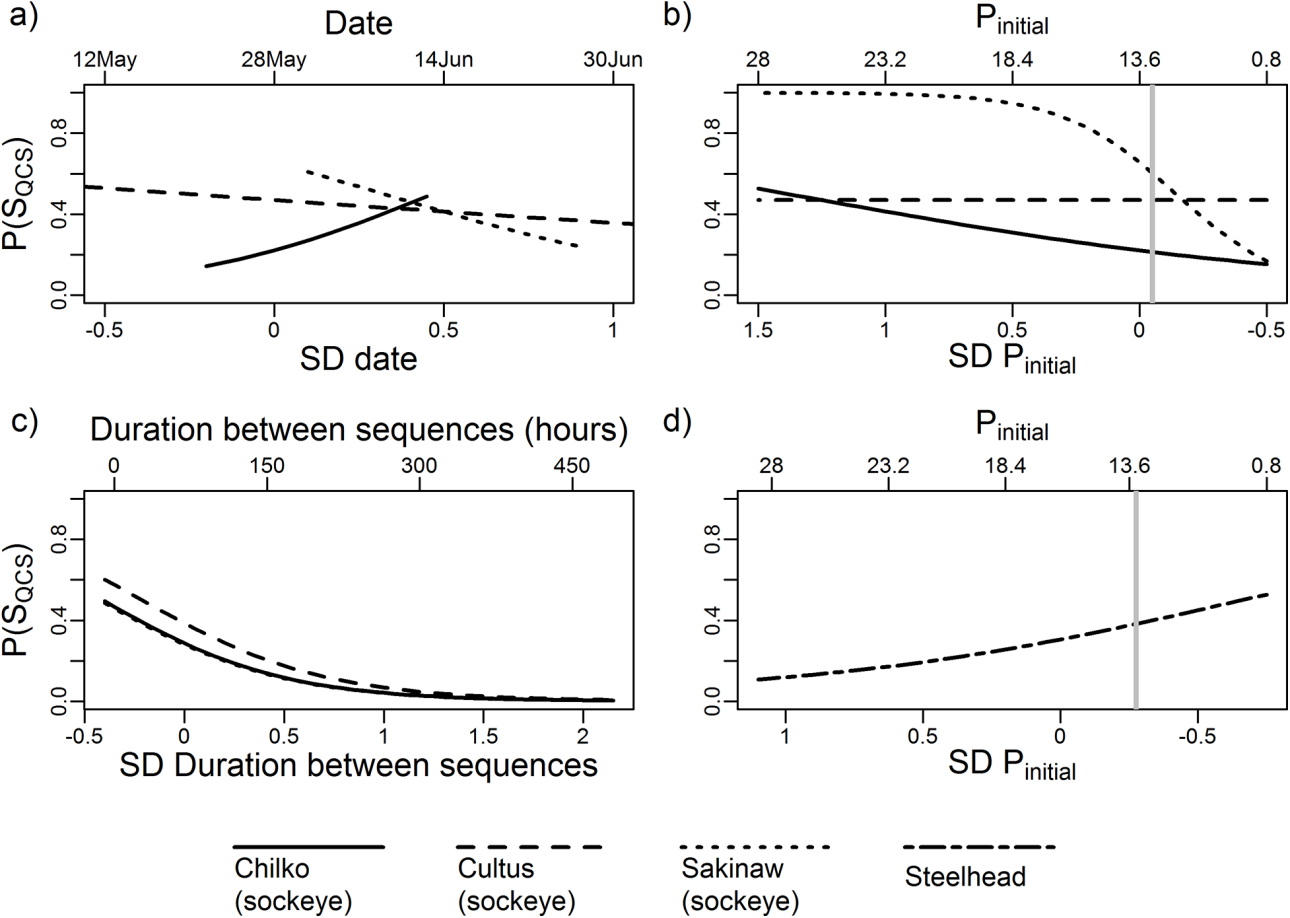
Prediction plots for the probability of survival to the Queen Charlotte Strait acoustic telemetry array [P(S_QCS_)]. Effects of (a) date of arrival at NSOG for sockeye, (b) receiver position of the initial detection sequence on NSOG (P_initial_) for sockeye, (c) duration between the first and second NSOG detections for sockeye, and (d) P_initial_ for steelhead. For all effects shown the 95% confidence interval of the model-averaged effect size did not contain zero (Fig 7). The upper x axis depicts the actual values of the variable, and the lower x axis are these values as standard deviations (SD) from the mean. For sockeye date of arrival (a), only the range of dates observed are predicted for each population. Vertical gray bars (panels b and d), indicate the separation of Malaspina Strait (receivers 1–7) from the Strait of Georgia (receivers 8–27). The predictions for survival of Chilko and Sakinaw sockeye across duration values (panel c) are very similar and therefore little separation of the prediction lines occur.

Model averaging of GLMs identified movement metrics that influenced probability of survival to the QCS array (Table 2, Fig 7). For initial arrival of sockeye to the NSOG array, the top-ranked model included all variables (initial position on the array, Julian date, natal population, and a population’s interactions with the preceding two variables), and was the only model for which ΔAICc ≤ 2 (Table 2). Chilko and Sakinaw sockeye smolts (Fig 1, Table 1) arriving via eastern NSOG receivers experienced poorer survival to QCS (Fig 8), with sockeye detected migrating west of Texada Island via the Strait of Georgia 1.4-times more likely to be detected at Queen Charlotte Strait than those detected migrating via Malaspina Strait, although these odds were not significantly different from one (Fisher’s exact test, P = 0.09). Sockeye smolts arriving via Malaspina Strait took less time to arrive to the NSOG array (p < 0.001), but these same smolts took longer to reach the QCS array among survivors (p = 0.02).

**Table 2:** Descriptions and summary statistics of all models with ΔAICc < 2 for all smolts detected at the NSOG array (A) and for all smolts with ≥ 2 detection sequences at the NSOG array (B) for both sockeye and steelhead smolts. ‘logLik’ is the log-likelihood, and W_i_ is the AICc weight of the model.

	Species	Model	R^2^	Adj R^2^	logLik	AICc	ΔAICc	W_i_
(A)	sockeye	P_Initial_ + Julian + Population + P_Initial_:Population + Julian:Population	0.11	0.15	-331.61	681.54	0.00	0.71
	steelhead	P_Initial_ + Julian + Year	0.05	0.07	-126.41	261.00	0.00	0.10
		P_Initial_ + Julian	0.04	0.06	-127.48	261.07	0.06	0.10
		P_Initial_ + Julian + Population	0.06	0.08	-125.49	261.26	0.25	0.09
		P_Initial_ + Julian + FL	0.04	0.06	-126.95	262.09	1.09	0.06
		P_Initial_ + Julian + Year + FL	0.05	0.08	-125.96	262.21	1.21	0.06
		P_Initial_	0.03	0.04	-129.13	262.31	1.30	0.05
		P_Initial_ + Julian + Year + Population	0.06	0.09	-125.09	262.58	1.57	0.05
		Julian + Year	0.03	0.05	-128.43	262.98	1.98	0.04
(B)	sockeye	Duration + Population	0.08	0.11	-86.66	181.60	0.00	0.30
		Duration	0.05	0.06	-88.97	182.03	0.43	0.24
		Duration + Population + Δ*x*_12_	0.08	0.11	-86.40	183.24	1.64	0.13
		Duration + Δ*x*_12_	0.05	0.07	-88.65	183.46	1.87	0.12
	steelhead	Duration	0.04	0.05	-36.18	76.57	0.00	0.12
		Duration + FL	0.06	0.09	-35.40	77.22	0.65	0.08
		Duration + FL + Δ*x*_12_	0.06	0.08	-35.48	77.37	0.80	0.08
		FL	0.02	0.03	-36.64	77.49	0.92	0.07
		Stock	0.01	0.01	-37.03	78.27	1.70	0.05
		Duration + Stock	0.04	0.06	-36.05	78.51	1.94	0.04

Chilko sockeye smolt survival also increased with Julian date of initial detection at NSOG, while both Sakinaw and Cultus populations demonstrated a negative relationship with Julian date of arrival (Fig 8). Although later Chilko smolts took longer to arrive to the NSOG array (p < 0.0001), among Chilko fish to survive to QCS, later migrants took less time to reach QCS (p = 0.05). For sockeye smolts with at least 2 detection sequences, the duration between NSOG detection sequences was retained in all models with ΔAICc ≤ 2, with longer durations decreasing survival probability (Fig 8).

For steelhead, both initial position along the NSOG array and Julian date of arrival were retained within all but one of the NSOG entry models (Table 2), but only for initial position did the 95% confidence interval of the model-averaged coefficients not contain zero (Fig 7). Unlike sockeye, no effects of natal population on survival were observed, despite Englishman River steelhead entering the Strait of Georgia from the western side (Vancouver Island) rather than from the eastern side (British Columbia mainland). Collectively, steelhead that migrated via Malaspina Strait were twice as likely to be detected at QCS than those that did not (Fisher’s exact test, P = 0.02). Steelhead arrival position was not correlated with migration speed either to the NSOG array (p = 0.97) nor the QCS array among survivors (p = 0.20). All of the variables within lateral movements models had 95% confidence intervals of model-averaged effects that contained zero, indicating overall weak relationships with subsequent QCS survival. However, the two variables “Duration between NSOG detections” and “FL” were included in multiple models with ΔAICc ≤ 2 (Table 2).

## Discussion

### Migration Routes

Although detections of smolts occurred across the entire NSOG array, both species preferentially migrated via Malaspina Strait, and extremely few (<2%) used the “westward” migration route through Juan de Fuca Strait, providing further support that upon exit from freshwater, most smolts move northward within the Strait [17,18,46] near the eastern coastline [18,19,29]. Juvenile salmon are thought to orient towards marine feeding grounds using a “magnetic map sense” [47,48] that would allow smolts entering the Strait of Georgia to orient to the northwest (i.e., through the Strait of Georgia rather than Juan de Fuca Strait). In addition, tendencies to migrate near shore have been observed in smolts across systems [18,49–51], a behavior that possibly facilitates fine-scale orientation and navigation [25] that would result in increased use of Malaspina Strait relative to the main body of water forming the Strait of Georgia. Quickly turning northward may also help to explain why the Englishman River steelhead stock did not display a disproportional use of Malaspina Strait, as their ocean migration initiated along the shore of Vancouver Island rather than the British Columbia mainland.

Approximately 40–60% of smolts of both species did not did not experience multiple bouts of geographically separate detections on the NSOG sub-array, and therefore may be moving in a relatively linear fashion through the Strait of Georgia. Linear movements are consistent with the current paradigm for smolt outmigrations presented by Groot and Cooke [18] that sockeye and steelhead exhibit relatively rapid coastal migrations [20,21,46]. The combination of a consistent migration heading (or “compass bearing”) and active swimming would help to facilitate such linear migration paths. Although these presumed-linear migrants were among the largest contingents for both species, it was surprising that more smolts were not classified as linear migrants given the mean speed at which migration occurs (~1 BL/s achieved speed over ground, or 13–17 km/day; [17,22]).

We present evidence that substantial contingents of both species (20–40% of sockeye and 30–50% of steelhead) experience westward or even fully counterclockwise movements while in the Strait of Georgia, particularly for steelhead. First, within initial detection sequences both species exhibit slight westward movements that indicate an angular trajectory across the array, although it should be noted that the array itself is slightly angled from the southwest to the northeast (Fig 1 inset). Second, a westward displacement was primarily observed between first and second detection sequences for both species, equating to westward movements of ~5km for sockeye and ~9.5 km for steelhead, with 25% of sockeye and >50% of steelhead experiencing lateral movements exceeding 8 km. As a result, the distributions of the subsequent detection sequences were more frequently recorded in the western Strait of Georgia, particularly for steelhead who rarely had subsequent detections in Malaspina Strait. It should be noted that steelhead had both greater prevalence and magnitude of westward movements even though steelhead used more western routes than sockeye initially, indicating that westward movements were not simply random. Third, westward displacements of steelhead were followed by a mean eastward displacement of ~1.5 km between the second and third detection sequences, indicating the potential presence of “loop-like” trajectories. Collectively, these observations indicate counterclockwise motions within the Strait of Georgia, with steelhead experiencing more pronounced counterclockwise movements than sockeye that appear to persist through three detection sequences that result in a net-transport of smolts from Malaspina Strait to the Strait of Georgia. This differs from previous research that suggested initial migrants use Malaspina Strait followed by use of the Strait of Georgia by later migrants that was based on purse seine surveys [18]. The westward transport we describe provides another potential mechanism for the observations by Groot and Cooke [18].

An organism’s migration route or trajectory can be influenced by the medium through which it is traversing [1], which is especially true for small-bodied organisms, such as salmon smolts, in marine environments [49,52]. The region is characterized by strong tidally-influenced currents in the northern passage (Discovery Passage/Johnstone Strait) as well as weaker currents in the central Strait of Georgia driven largely by tide and freshwater discharge, including the region of the NSOG array [53–55]. Estuarine flow results in a net northward flow in the surface layer over the tidal cycle in the central-to-northern Strait [53,55], and these tidal currents are thought to influence outmigration routes of smolts [18,19]. Due to the shape and structure of the central Strait of Georgia, the current direction at any given location turns counterclockwise throughout the tidal period, such that rotary tidal currents develop [53]. Furthermore, fine-scale ocean simulation models predict areas of overall counterclockwise circulation in the region extending from the NSOG array northward [54]. In particular, the region of the NSOG array exhibits mean surface currents flowing northward through Malaspina Strait and much of the Strait of Georgia west of Texada Island, and then strong mean currents flowing southward along the most western 3–5km of the Strait [54]. We propose that these currents contribute to the counterclockwise movements observed in both sockeye and steelhead. Although we did not find the same evidence for such movements at the QCS array (not shown), sample sizes at this site were limited due to smolt losses from mortality. However, it certainly seems possible that similar circular smolt movements will occur elsewhere where tidally-rectified eddies develop.

Juvenile smolts are larger and stronger swimmers than the larval fish which are often the focus of marine connectivity studies (i.e., [52,56]). In fact, simulation studies have suggested that smolts in nearshore environments cannot act as passive particles to complete known migrations [19,49]. Therefore the movement behaviors of smolts should play a large role in defining overall migratory trajectories or routes [49], although the balance between oceanography and self-directed movements is largely unknown [21]. Movement behaviors encompass swim speed, swim depth, the use of navigation and/or orientation capabilities, and fine-scale responses to variability in resources, gradients in water quality, and/or predators (many of these factors reviewed in [25]). It is possible that the use of navigation and/or orientation capabilities in the Strait of Georgia allows smolts to orient towards the northwest (thus exiting the Strait of Georgia and facilitating movement towards presumed marine winter feeding areas in the offshore North Pacific [47]), but that the ability to maintain linear routes is limited by oceanographic currents and/or swimming capabilities.

In particular, swimming depth has the potential to influence the trajectory of migrating salmon smolts [25,49]. Smolts are believed to swim in shallow near-surface waters [57,58]. Although currents in the region are dominated by tidal flow [53,54], northwesterly winds are present during the spring and summer migrations of smolts that can modify currents at the surface [53,55]. These winds generally act to decrease the mean northward flow of surface waters in the central-to-northern Strait, but this influence decays with depth via the processes creating Ekman spirals [59], which have been observed in the Strait of Georgia [60]. Thus, variability in swim depth among individuals and between populations and species would alter the exposure and relative impact of wind-altered surface currents on migratory trajectories. In freshwater, juvenile steelhead have been found to swim at shallower depths (<3 m) than other salmonids [61], and in the pelagic offshore environment of the open ocean immature steelhead swim much closer to the surface than the other species of Pacific salmon, based on the relative vertical distribution of species-specific catches in gill nets (DWW, unpublished observations). Steelhead swimming at shallower depths than sockeye provides a potential mechanism for the observed increase in westward transport of steelhead within the Strait of Georgia and the increase in the measured magnitude of counterclockwise movements relative to sockeye (Figs 5 and 6). Unfortunately, depth-sensing acoustic tags still remain too large to implant into smolts, and therefore it is unknown what patterns may exist in swim depth across individuals and species.

In addition to swim depth, the relative impact of currents on the migration trajectory is dependent upon individual swim speed of smolts. In general, migration rates (speed over ground) of smolts approach 1 BL/s across species ([17,22]). If size-dependent swim speeds are consistent, larger fish would be expected to have increased overall swim speeds and therefore be proportionately less influenced by surface currents. We did not observe any differences, however, in the overall magnitude of western movements across populations of sockeye smolts, even though Cultus sockeye smolts were generally 40% longer (~50 mm) than Chilko smolts. In addition, steelhead experienced larger westward movements than sockeye even though they were generally of the same size (or larger). Thus size differences of this order might not be of measurable influence on migration behavior and/or routes. In fact, more robust migration speed analyses on a similar dataset found that body size did not have a large impact on straight-line migration rates [17].

Caution is needed, however, when interpreting straight-line estimates of migration rates, as they do not account for complex movement behaviors such as those we have observed in this study. In fact, due to the prevalence and magnitude of lateral movements we observed in both species, we suggest that straight-line estimates of migration speed (speed over ground) may substantially underestimate true swimming speeds. We cannot provide further information regarding actual swim speeds, however, as we are unable to determine the relative influence of swim speed and current speed on overall migration rate given the current resolution of marine telemetry arrays. The development of high-resolution individual-based models (IBMs) to simulate movements within a realistic oceanographic environment can be used to test hypotheses regarding the balance between swim speeds, movement behaviors, and ocean currents (i.e., [25,49]), while the use of fine-scale 3D tracking arrays could provide direct measurements of the degree of tortuosity in at least some environments (e.g., [32,62]).

The ability to determine migratory behaviors or routes in the current study is dependent upon the NSOG array’s ability to detect smolts as they migrate through. V9 tags have an estimated 90% detection efficiency at the NSOG array, while smaller V7 tags have an estimated 60–75% efficiency [29]. Imperfect detection efficiency will result in underestimating the extent and nature of rotary movements, as 1/3^rd^ of the first detections of individual V7 tags likely actually represents the second visit to the array. Thus a significant fraction of the apparently clockwise movements recorded (first detection to the west of the second set of detections) may actually come about because the initial point of contact of V7 tagged smolts with the NSOG array went undetected (a similar argument applies to the 2^nd^ and 3^rd^ bouts of detection on the array). These differences in detection efficiency among tag types complicate our comparisons among sockeye populations and between the two species, as all steelhead and Sakinaw and Cultus sockeye were implanted with V9 tags where this “aliasing” effect will be substantially less, while Chilko sockeye were implanted with smaller V7 tags. Although these differences exist, and individual variability in migratory experience was observed, it is evident in the current study that the behaviors described occur across populations and in both species, and thus appear to be common among smolts rather than anomalous observations.

### Survival

Smolt survival in the Strait of Georgia to QCS varied among initial NSOG entry positions for both species, suggesting that conditions important to the migratory process are spatially variable in the Strait of Georgia. Post-release survival was highest for steelhead arriving along western receivers, even though steelhead disproportionately arrived via Malaspina Strait to the east. Steelhead arrival position was not correlated with migration speed either to the NSOG array nor the QCS array among survivors and thus we don’t expect this result to be confounded by differences in migration rates among routes taken. Therefore the specific migration route taken by steelhead through the Strait of Georgia appears an important part of the migration process.

For sockeye, although the 95% interval of the effect size narrowly included zero, Chilko sockeye also experienced poorer survival to QCS when arriving via Malaspina Strait. Among sockeye populations, only for Sakinaw smolts did the 95% interval of the effect size exclude zero. Of the three sockeye populations investigated, Sakinaw had the lowest sample size, and the release point for Sakinaw sockeye lies within Malaspina Strait, quite close to the eastern receivers (Fig 1), and therefore we limit our interpretations of this finding. Future studies should focus on identifying the factors that influence smolt survival on finer spatial and temporal scales to better understand why migration routes affect smolts, and why the observed relationships between migration routes and survival may differ between species.

Texada Island (separating receivers 7 and 8; Fig 1 inset) is a natural barrier that could contribute to variable conditions experienced by smolts. The factors that could vary spatially in the Strait and influence smolts are numerous. Variability in currents and zooplankton could influence both swimming and feeding efficiency. Zooplankton communities have been assessed on longer (decadal) time scales [63,64] but spatiotemporal variability within the spring and summer in the Strait of Georgia is very difficult to assess at present (but see [65]). Feeding and energy use affects smolt growth, which has been suggested to influence coastal survival by altering predation risk along the migration [11,12]. Although research has identified individual taxa that predate on smolts (e.g., seals [66], dogfish [67], lamprey [68]), the spatial and temporal distribution of these predators, and which predators have the biggest impacts on specific species of smolts, are largely unknown. Further studies should aim to better understand the movement, distribution, and behavior of smolt predators, as well as utilize technologies capable of examining predator-prey interactions directly (i.e., [69]).

In addition to the initial migration route used, the timing of arrival at the NSOG array was identified as an important variable in predicting subsequent sockeye survival. Arrival date was also retained in nearly all of the top models for steelhead, but the effect was weaker and the 95% confidence intervals of its effect size contained zero. Later migrants for Cultus and Sakinaw sockeye experienced poorer survival but examining the prediction plots reveals that these effects were quite weak. Later arriving wild Chilko migrants, however, experienced improved survival. Salmonid migrations are generally thought to occur within specific time periods as a result of evolutionary selection [70–72]. Timing has repeatedly been found to influence migratory success of adult salmon during spawning migrations (e.g., [73–75]), but examples for juvenile smolts are less common (but see [76,77]). Satterthwaitte et al., [77] demonstrated improved survival of juvenile hatchery Chinook released 70 to 115 d after the spring transition, indicating potentially important bottom-up influences. Scheuerell et al., [76] found increased survival for earlier migrants of juvenile Chinook and steelhead outmigrating from the Columbia River system, which they attributed to temporally variable nearshore environmental conditions and predator distributions. In particular, regional-scale oceanographic conditions, including temperature and upwelling indices [78,79], have been correlated with early marine survival of smolts. These studies, however, focus on interannual variability rather than changes in intra-seasonal conditions that our results suggest might be important. Future work should attempt to characterize the differences in migration experience among outmigration dates of smolts. Regardless, the importance of timing suggests that factors influencing survival not only vary spatially but also temporally within the Strait of Georgia.

Sockeye smolts with longer durations between the first two detections sequences experienced poorer survival to QCS, while this effect was found to be weaker for steelhead, such that the 95% confidence interval of the effect size contained zero. Furthermore, for sockeye both initial position and migration timing were correlated with migration rate for those surviving to QCS, such that migration rate may explain the identification of these variables as important. Combined, our results suggest that smolts milling near the array for extended periods of time can experience poorer survival and that sockeye survival in the Strait of Georgia is proportional to time at some level. Even though sockeye survival appears time-dependent, it should not be ignored that migration timing and route resulted in varying durations to reach QCS, and thus variability in route still has an influence on smolt experience within the SOG. Given that duration between detections sequences at NSOG was correlated with the size of the lateral movements, it is still difficult to assess how strongly survival is linked to time rather than distance travelled. Regardless, excessive milling of smolts presumably decreases survival at least in proportion to the longer time spent within the region monitored by the array.

Growth and/or size of migrant smolts has been implicated as an important process in determining survival during the marine migration [11,12,80], as larger smolts should have increased swim speeds to better evade predators and have fewer gape-limited predators. Although FL was not included in sockeye models (due to collinearity with population), we were still surprised that FL had little support across steelhead models, and confidence intervals of the estimated effect of FL always included zero. This apparent lack of importance of smolt size could be a result of the narrow size ranges of tagged fish, as well as the largest fish in populations being selected for acoustic tagging for both species (the standard deviation of FL within steelhead release groups was ≤17 mm, and ≤15 mm for sockeye). In addition, Cultus hatchery sockeye were ~100 mm larger than their wild conspecifics [29] and of Chilko fish, only the larger age-2 smolts could be tagged, which in general make up <10% of the outmigrant population [81] and are ~50 mm larger than age-1 smolts. Therefore it is possible that our narrow range of sizes tagged, relative to the broader population, prevented our ability to detect size-specific differences. Irvine and Akenhead [81], however, found no difference in average smolt-to-adult survival rate for age-1 and age-2 wild Chilko sockeye smolts over a ~50 year time series. Nevertheless, it is interesting that migration route-related factors were found to be more tightly linked to survival than body size.

In summary, our ability to link migration metrics to subsequent survival at a location ~250 km away is promising evidence of the power of sophisticated marine telemetry arrays to make important (and previously impossible) measurements of the importance of migration routes and movement behaviors on the migratory process. Using individual-based telemetry, we have characterized smolt movements in the marine environment at previously undescribed spatial scales and temporal resolutions. Our results confirm that smolts use Malaspina Strait disproportionately, but although most smolts move linearly through the NSOG array, both species demonstrate substantial contingents of individuals (~25–50%) that move in westward or counterclockwise pattern that result in a net transport of smolts from Malaspina Strait to the Strait of Georgia, especially steelhead. Counterclockwise movements may be due to the predominate currents in this area during the time of outmigration, as well as biological differences in swimming behavior. Beyond identifying the main migration routes and characterizing variability in these routes among smolts, links were established between survival and characteristics of the migration, including initial migration route and timing, and the duration of time spent in the Strait of Georgia, suggesting that the factors important to migratory success are both spatially and temporally variable within the Strait of Georgia. In particular, steelhead appear affected by migration route regardless of migration rate. Our results provide a rare empirical example for how movements can affect survival within the field of movement ecology [1,10], and confirm that variability in movements are an important part of the migratory process.

## Supporting Information

S1 Fig Scatterplot of the relationship between the duration (in hours) between the first and second detection sequences and the difference in receiver position (km) for sockeye and steelhead smolts at the northern Strait of Georgia (NSOG) array. Best-fit regression lines for each species are added for visualization. Points are jittered by up to 0.5 units horizontally to aid visibility. Correlations were significant for both sockeye (Kendall’s τ = 0.49; P < 0.0001) and steelhead (Kendall’s τ = 0.38; P < 0.0001) smolts.
(TIF)
