# Supplementary figures and images for: Variability in Migration Routes Influences Early Marine Survival of Juvenile Salmon Smolts
Source: PLoS One. 2015 Oct 9;10(10):e0139269. doi: 10.1371/journal.pone.0139269 (PMC4599731; doi:10.1371/journal.pone.0139269)

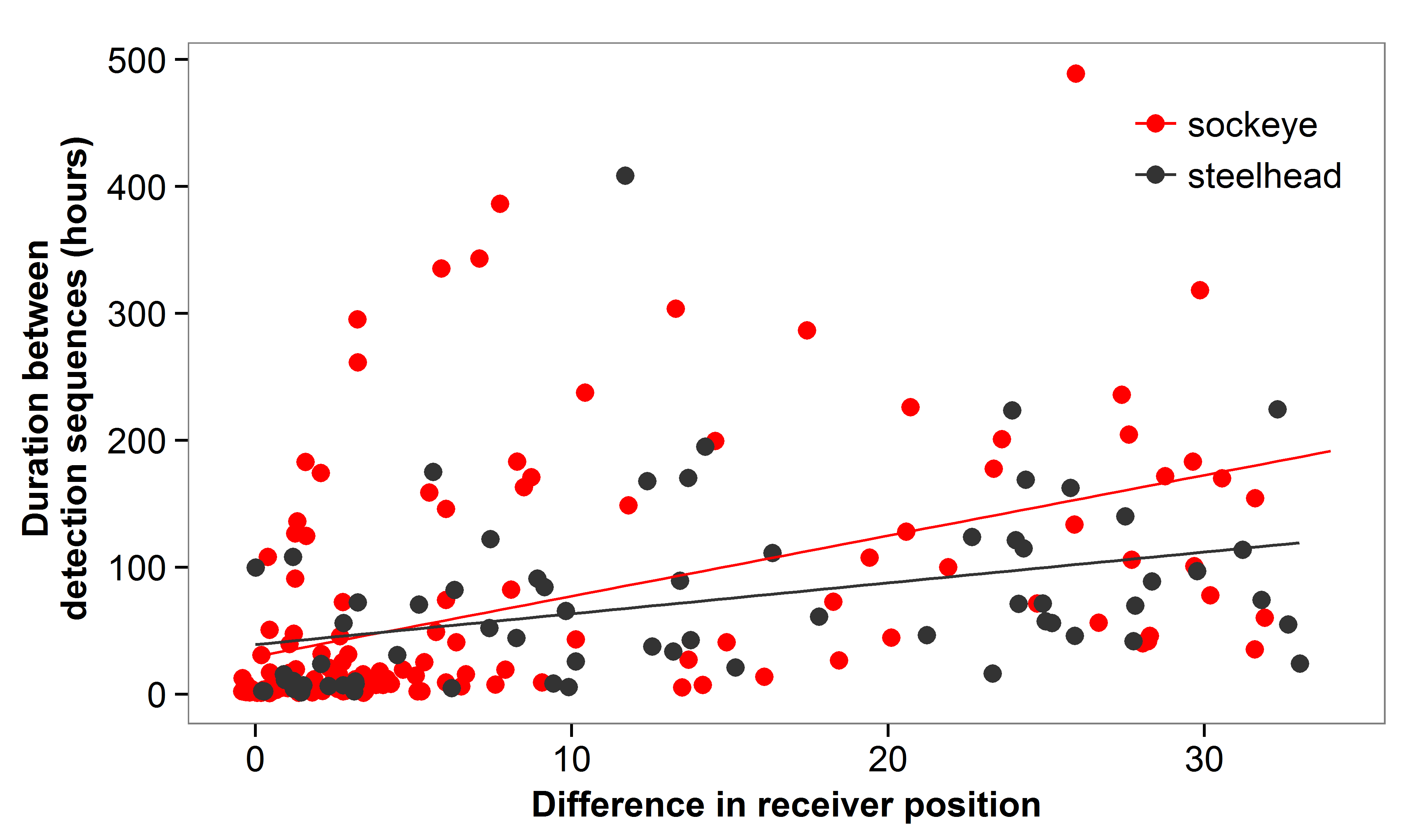

Supplement: S1 Fig — Best-fit regression lines for each species are added for visualization. Points are jittered by up to 0.5 units horizontally to aid visibility. Correlations were significant for both sockeye (Kendall’s τ = 0.49; P < 0.0001) and steelhead (Kendall’s τ = 0.38; P < 0.0001) smolts. (TIF) [file pone.0139269.s001.tif]
